# GATA 2 Deficiency: Focus on Immune System Impairment

**DOI:** 10.3389/fimmu.2022.865773

**Published:** 2022-06-13

**Authors:** Francesco Fabozzi, Angela Mastronuzzi, Giulia Ceglie, Riccardo Masetti, Davide Leardini

**Affiliations:** ^1^ Department of Hematology/Oncology, Cell and Gene Therapy, Bambino Gesù Children’s Hospital, Rome, Italy; ^2^ Department of Pediatrics, Università degli Studi di Roma Tor Vergata, Rome, Italy; ^3^ Pediatric Oncology and Hematology “Lalla Seràgnoli”, IRCCS Azienda Ospedaliero-Universitaria di Bologna, Bologna, Italy

**Keywords:** GATA2 deficiency, transcription factors, inborn errors of immunity, recurrent infection, autoimmune disease, myelodysplastic syndromes, hematopoietic stem cell transplantation

## Abstract

GATA2 deficiency is a disease with a broad spectrum of clinical presentation, ranging from lymphedema, deafness, pulmonary dysfunction to miscarriage and urogenital anomalies, but it is mainly recognized as an immune system and bone marrow disorder. It is caused by various heterozygous mutations in the *GATA2* gene, encoding for a zinc finger transcription factor with a key role for the development and maintenance of a pool of hematopoietic stem cells; notably, most of these mutations arise *de novo*. Patients carrying a mutated allele usually develop a loss of some cell populations, such as B-cell, dendritic cell, natural killer cell, and monocytes, and are predisposed to disseminated human papilloma virus and mycobacterial infections. Also, these patients have a predisposition to myeloid neoplasms, including myelodysplastic syndromes, myeloproliferative neoplasms, chronic myelomonocytic leukaemia. The age of symptoms onset can vary greatly even also within the same family, ranging from early childhood to late adulthood; incidence increases by age and most frequently clinical presentation is between the second and third decade of life. Currently, haematopoietic stem cell transplantation represents the only curative treatment, restoring both the hematopoietic and immune system function.

## Introduction

GATA family includes six nuclear transcriptional factors sharing the ability to bind to the consensus DNA sequence T/A (GATA)A/G; of those, GATA4, GATA5, and GATA6 are involved in cardiac formation, GATA3 is expressed in several tissues, whereas GATA1 and GATA2 have a leading role in hematopoiesis (1).

In the past decade, GATA2 deficiency has been recognized to cause previously distinct clinical entities, namely DCML deficiency (dendritic cell, monocyte, B, and NK lymphoid deficiency) (2), MonoMAC syndrome (monocytopenia with Mycobacterium avium complex infection) (3), familial myelodysplastic syndrome (MDS)/acute myeloid leukemia (AML) (4), Emberger syndrome (MDS with lymphedema) (5), and NK deficiency (6, 7). This wide spectrum of clinical manifestations highlights the complex role of this transcription factor: crucial for hematopoietic stem cell (HSC) activity and self-renewal, but also important for vessels and ear development (1, 8). Due to a very high lifetime risk for hematological malignancies, patients with GATA2 deficiency are candidates for hematopoietic stem cell transplantation (HSCT), currently the only current curative treatment. However, given the wide range of disease severity and the not negligible treatment related mortality (TRM), the optimal transplantation timing is still unclear (9). Herein, we briefly review the genetic and clinical features of GATA2 deficiency, focusing on immunological phenotypes. Finally, we summarize the state-of-the-art management options, shedding light on the current main research gaps.

## GATA2: Structure, Function, and Mutations

**Figure 1:**
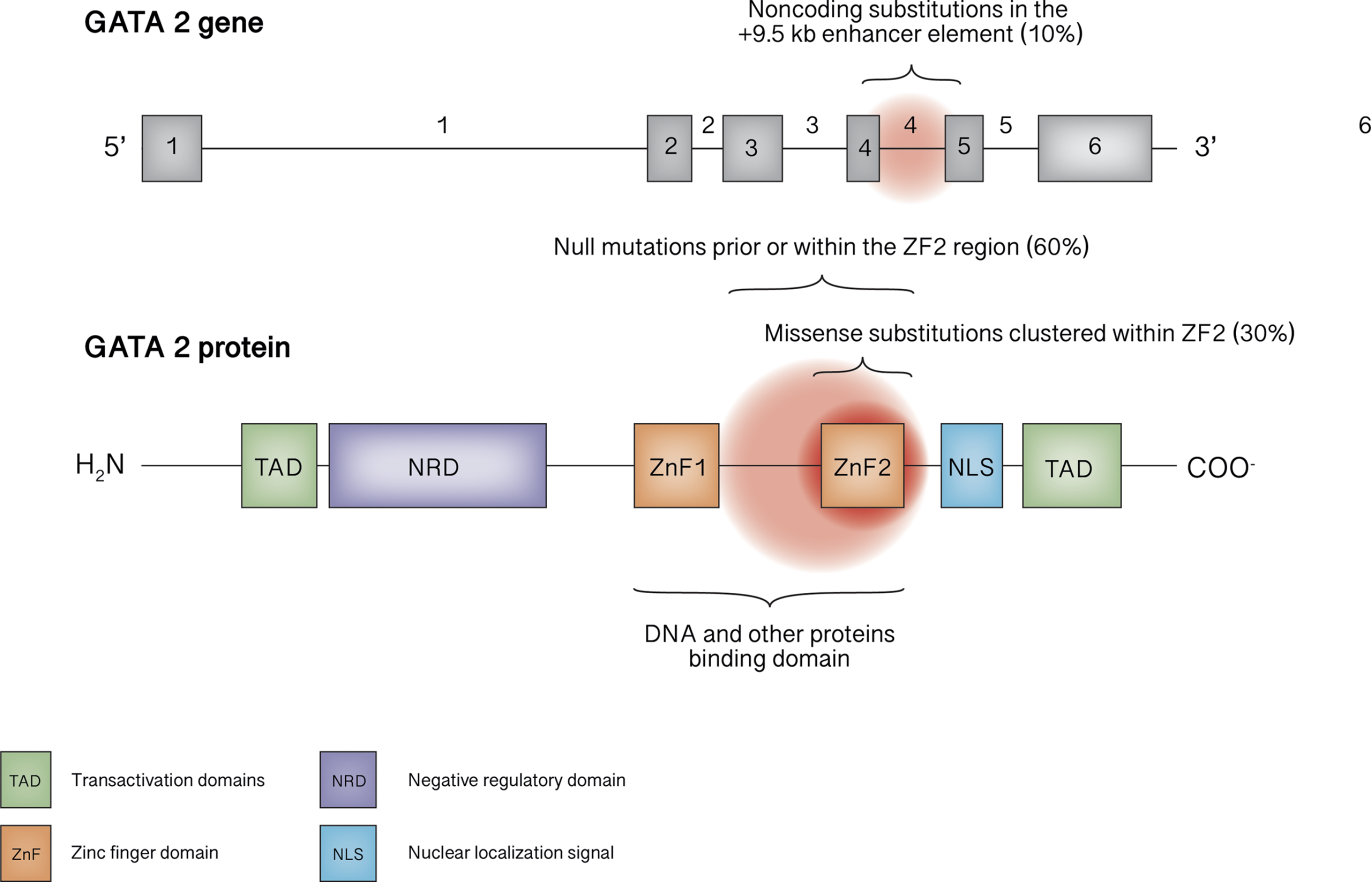
GATA2 gene and protein structure. The mutational spectrum in GATA2 deficiency mainly involves truncating mutations occurring before or within ZF2 or missense mutations within ZF2, as well as noncoding mutations within the +9.5 kb enhancer element.

The structure of this important transcription factor consists of two highly conserved zinc finger domains (ZF1 and ZF2), essential for binding to DNA and other proteins, a nuclear localization signal, two transactivation domains, and a negative regulatory domain (1) (**Figure 1**). While it can also be expressed in endothelial cells, central nervous system, placenta, fetal liver and fetal heart (10, 11), GATA2 plays a leading role in hematopoiesis through a complex network of interactions with other transcription factors such as RUNX1, SCL/TAL1, MYB,GFI1, FLI1,LYL1, or PU.1 (11). In fact, the homozygous knock-out in mouse embryo is lethal, due to failure in the endothelial-hematopoietic transition that produces the first adult HCSs (12). Of note, murine models with homozygous mutations that leave a residual but insufficient protein activity result in embryo death at a later stage, due to endothelial failure instead of the hematopoietic failure, highlightening the importance of gene dose (13). In post-natal life, GATA2 is essential for the maintenance of HSCs pool and for progenitor cell differentiation, for instance, mice carrying heterozygous GATA2 mutations exhibit a reduced amount of HSCs (11). Differently from human GATA2 deficiency phenotype, mouse model with GATA2 haploinsufficiency present normal bone marrow cellularity and peripheral blood cell count without the development of MDS or AML, suggesting a different effect of the mutation (14), probably as a consequence of the shorter mice lifespan (10). Thus, establishing a mouse model to study the mechanisms underlying this disease represents a current research challenge. With the aim of investigating whether further reduced expression of GATA2 could serve as a model for human disease, a hypomorphic mutant mouse line was established by Hoshino and colleagues, in which the expression level of GATA2 was reduced to approximately 20% of normal (15). Despite being apparently healthy at birth, approximately 70% of these mice die early due to bilateral ureterovesical junction obstruction, highlightening the role of gene dose in urogenital organogenesis (15). However, so far no case of death due to fatal urogenital malformations has been reported in humans, although urogenital malformations may occur in a certain proportion of people affected by GATA2 deficiency (16). Interestingly, mice that survive to adulthood are predisposed to develop myeloproliferative neoplasms resembling human chronic myelomonocytic leukemia (17). Nevertheless, distinctive features of GATA2 deficiency, namely cytopenia involving dendritic cells, monocytes, B or NK lymphocytes, were not found in this mouse line, indicating that quantitative deficits of GATA2 do not represent a suitable model of human disease. Recently, a very promising approach to this problem by following the qualitative rather than quantitative deficit way has been published by Hasegawa and colleagues (18). They generated mice that expressed the mutant GATA2 protein in which arginine 398 was substituted for tryptophan, resulting in a dominant-negative effect on the native protein in terms of DNA-binding activity that induces perturbations of the regulation of specific target genes. Mice were found to develop an age-progressive multilineage cytopenia of dendritic cells, monocytes, B and NK lymphocytes, resembling the human phenotype. However, the mice never developed hematopoietic malignancies, indicating that improvements are still needed in establishing a suitable model of GATA2 deficiency.

The *GATA2* gene is located on chromosome 3q21.2. To date, more than 150 pathogenic or likely pathogenic germline variants are reported, affecting nearly 500 individuals (11, 16, 19, 20). Null mutations (frameshift, nonsense, splice site and whole gene deletions), located prior or within the ZF2 region, underlie roughly 60% of cases, whereas missense substitutions clustered within ZF2 represent about 30% of mutations. Noncoding substitutions in the +9.5 kb enhancer element (EBOX-GATA-ETS regulatory region in intron 4) are responsible for 4-10% of cases; synonymous mutations introducing new splice donor site, that causes premature translation termination associated with nonsense-mediated decay, account for 8% of cases in the European Working Group of MDS in Childhood (EWOG-MDS) study (21). In addition, tandem GATA2 duplication was recently described in a patient with atypical mycobacterial infection who exhibited an increased transcription of the anti-sense long-non-coding RNA GATA2-AS1, probably responsible for the pathogenetic mechanism (22). Finally, germline mutations involving ZF1 have been described only rarely (23, 24).

Together, all mutations are loss-of-function and result in haploinsufficiency. How reduced levels of GATA2 can cause such a broad spectrum of clinical phenotypes, as well as variable penetrance, even within the same family, is not yet fully elucidated (25). The high variety of mutations could play a role, while epigenetic changes could underlie the reduced penetrance in asymptomatic patients (25–27).

## Clinical Features: Immunodeficiency, Bone Marrow Failure, and More

**Figure 2:**
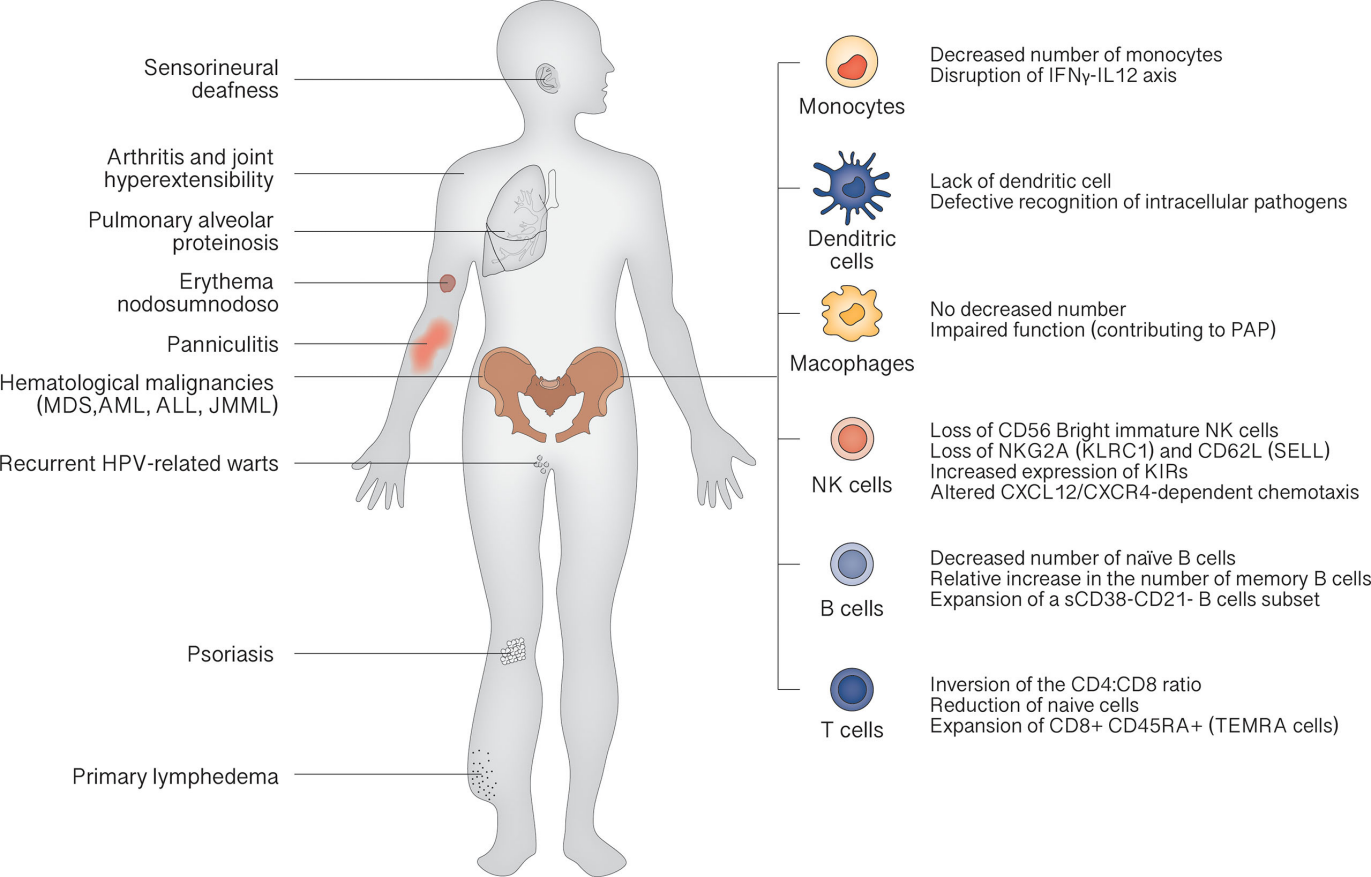
Clinical features of GATA2 deficiency.

The clinical landscape of GATA2 deficiency is dominated by bone marrow dysfunction, leading to recurrent infection (especially atypical mycobacterial infections and recurrent HPV-related warts) and hematological malignancies. Other characteristic features are pulmonary alveolar proteinosis (PAP) and lymphedema. The median age of clinical onset is 18 years, and the penetrance is estimated at 90% by the age of 60 years (28). At birth, patients show normal cell counts, as demonstrated in carriers recognized by familiar screening. Then, a progressive reduction of CD34+ cells in bone marrow can be demonstrated, inversely correlated with the serum levels of FLT3 ligand, a potential marker for monitoring the evolution of bone marrow failure (29). This leads to the classic peripheral blood cells decrease: monocytopenia, dendritic cell deficiency, NK cell deficiency, B cell deficiency and, less commonly, neutropenia. The severity of manifestations depends on peripheral cell counts: the more severe the cytopenia, the more severe the symptoms (**Figure 2**).

According to 2019 Update of the IUIS Phenotypical Classification, GATA2 deficiency is classified among congenital defects of phagocyte, underscoring the role of monocytopenia as a key feature (30). Lack of monocytes results in characteristic susceptibility to non-tuberculous mycobacterial infection, *via* disruption of IFNγ-IL12 axis that plays a crucial role in resistance to these microorganisms (31). In fact, these infections occur in about half of the cases in the study by Spinner et al. (28). Also lack of DCs contributes to mycobacterial susceptibility by defective recognition of intracellular pathogens. Despite the absence of monocytes, macrophages can be detected in lungs of GATA2 patients, but their function is probably impaired, concurring to PAP trough a not still elucidated mechanism (10, 32).

Once marrow failure is established B and NK precursors are not detectable in the bone marrow of GATA2 patients (33). Loss of CD56 ^Bright^ immature NK cells and skewing toward a more highly differentiated phenotype, with loss of NKG2A (KLRC1) and CD62L (SELL) and increased expression of killer cell immunoglobulin-like receptors (KIRs), are typical findings (29). The few circulating NK cells also present an altered CXCL12/CXCR4-dependent chemotaxis (7, 34). A decrease in the number of naïve B cells, with a relative increase in the number of memory B cells and an expansion of a small subset of CD38-CD21- B cells associated with autoimmunity, is constantly found (29).

Although less compromised than other lymphocyte subsets, T cells show inversion of the CD4:CD8 ratio, with a reduction of naive cells and expansion of terminally differentiated CD8+ CD45RA+ effector cells (TEMRA cells). Taken together, the cellular phenotype resembles the pattern of terminal differentiation seen in age-related immunosenescence or in chronic viral infections (35).

The above-described alterations of immune system account for the high rate of infectious diseases in GATA2 patients, HPV and atypical mycobacterial being the most commonly found pathogens. Recurrent warts are the most common presentation of HPV infection, occurring in about half of patients, and are typically refractory to treatment (23, 28). The exact mechanisms of control of HPV infections are unclear, but cellular and cytotoxic immunity may have a crucial role. Both CD4 and CD8 T cells are found in regressing genital warts, and there are increasing evidences about the importance of CD4 T cell responses to E2 and E6 specific HPV proteins in controlling HPV-16 (36). The defective immunosurveillance by NK cells is responsible for a high rate of malignant transformation of HPV lesions. In addition, a significant amount of patients suffers from severe herpes virus infections, occurring in about 1/3 of patients: recurrent stomatitis, esophagitis, genital infections due to HSV 1-2, as well as severe VZV, EBV and CMV infections (28). Reduction of naïve B and T cells, with reduced NK cell-mediated cytotoxicity, promotes severity of EBV infections, as well as neoplastic transformation by the virus; moreover, the ability of the EBV to maintain latency may be disrupted by reduced expression of NOTCH1, TRAF2, and TRAF3 in cells from patients with GATA2 deficiency, resulting in increased virus replication (37). Finally, severe fungal infections (aspergillosis, histoplasmosis and candidiasis) occur in approximately 16% of patients (28).

Recently, rheumatological manifestations were found in 17.8% of patients in a study (38), and a correlation with a lower CD4:CD8 T cells ratio was found. Surprisingly, other than panniculitis, erythema nodosum, psoriasis and arthritis, non-immunologically mediated disorders such as piezogenic pedal papules and joint hyperextensibility were found in a significant number of patients, suggesting a role of GATA2 in collagen architecture. This is a distinguishing feature of GATA2 deficiency, since non-immunologically mediated rheumatologic manifestations have not been described in MDS patients carrying wild type GATA2 (39, 40). On the other hand, autoimmune manifestation can be found also in non-GATA2 mutated MDS with a similar prevalence, although other differences exist between the two phenotypes. In non-GATA2 mutated MDS rheumatologic disorders usually develop months/years after MDS diagnosis, whereas in GATA2 deficiency they can develop independently from hematological manifestations even without any other clinical symptom (40). Moreover, the most prevalent rheumatologic manifestations in the non-GATA2 mutated MDS group are vasculitis, namely polyarteritis nodosa and giant-cell arteritis, differently from GATA2 mutated patients (39, 40).

Although immunodeficiency represents one of the main features of the clinical picture, the high risk of developing myeloid neoplasms is the main sword hanging over the head of patients with GATA2 deficiency, presenting in more than 80% of patients by the age of 40 (41). The mechanism underlying this risk is likely to be found in the continuous stress, due to cytopenias and recurrent infections, to the malfunctioning bone marrow, leading to an accumulation of somatic mutations and selection of a clone with proliferative advantage in a predominantly hypocellular environment; ASXL1 alterations drive most frequently leukemogenic transformation (42, 43). Together with *SAMD9/9L* mutation, *GATA2* germline mutation is one of the most common genetic cause of MDS, causing 15% of advanced forms and 7% of all primary MDS in childhood (44, 45). Mirror wise, MDS is the commonest malignancy among this population, representing roughly 3/4 of neoplasms, followed by AML, that can evolve from MDS or be the initial presentation (23, 41, 44). Lymphoblastic acute leukemia (ALL), juvenile myelomonocytic leukemia (JMML), and aplastic anemia (AA) can also occur (23, 41, 44). Cytogenetic aberrations are frequently identified in GATA2-related MDS: monosomy 7 or der(7; 1q) are the most common, found in about 41% of cases, followed by trisomy 8, identified in roughly 15% of cases among published studies (11, 20); moreover, complex karyotypes are generally very rare, whereas del5q have never been described (11, 20). It should be emphasized that monosomy 7 in pediatric MDS is highly suggestive of a genetic alteration, with a germline mutation of SAMD9/9L or GATA2 found in about half of the cases (45). Notably, a GATA2 mutation is not a negative prognostic factor among MDS (44, 46).

Other clinical features characteristic of GATA2 deficiency are lymphedema and sensorineural deafness, showing the crucial role of GATA2 in the development of both lymphatic vessels and lymphatic valves, as well as of semicircular canals and perilymphatic spaces (47, 48). GATA2 patients also have an increased risk of neoplasms other than hematologic, likely caused by impaired immunosurveillance (10). Miscarriages are frequently reported and are probably due to NK lymphopenia (28, 49).

## Current Management and Future Perspectives

Given the broad spectrum of clinical manifestations, the diagnosis of GATA2 deficiency can be challenging. A strong clinical suspicion must arise when characteristic features, such lymphedema, deafness, recurrent HPV-related warts, PAP, and atypical mycobacterial infection, are present together with cytopenia including monocytopenia alone. Moreover, all children with high-risk MDS should be considered for GATA2 deficiency, especially those affected by MDS with excess blasts (44, 45). Intrafamilial recurrence of MDS may be another important clue. Once the mutation is found, a baseline bone marrow biopsy should be performed, and all family members should be tested given the variable penetrance. Because of progressive nature of this disease, healthy carriers must be warranted a close follow-up, including complete blood count, lymphocyte subset assessment, bone marrow aspiration (with flow cytometry, cytogenetic and mycobacterial culture), complete dermatologic examination, pulmonary function tests and, in women, gynecologic examination (41, 42, 50). Routine vaccinations should be administered; live virus vaccinations are safe as long as blood counts are normal, while there are no data regarding cytopenic patients (50). Azithromycin prophylaxis could be considered as blood counts decrease, although its use is not supported by consistent evidence (42).

Currently, HSCT represents the only curative treatment, as it has been shown to restore normal hematopoiesis, resolving MDS, clearing underlying infections and improving pulmonary function. However, the great variability in clinical presentation as well as age of onset raises several questions about who and when to transplant. This decision is usually driven by occurrence of malignancies in childhood, and by both malignancies and life-threatening infections in adulthood. Recently, HSCT outcome was demonstrated to be independent of *GATA2* germline mutations in a EWOG-MSD study regarding pediatric MDS, suggesting the application of standard MDS algorithms and protocols in this population (46). On the other hand, a better prognosis was found when HSCT is performed early in the disease: in fact, since GATA2 related MDS tends inexorably to progress in more aggressive forms such as leukemia, may be wise considering a preemptive HSCT (46). The excellent HSCT outcome was also confirmed by Nichols-Vinueza et al. in in a different cohort of adult patients (mean age about 28 years) underwent HSCT for life-threatening infections or MDS. The reported overall survival (OS) and event-free survival (EFS) at 4 years were 85% and 82% respectively (51).

Further research is needed to find other effective treatments other than HSCT. Gene editing technologies, such as CRISPR/Cas9 system, representing one of the most intriguing approaches, in the wake of the success achieved in the treatment of other hereditary hematological diseases (52).

Since a heterogeneity of mutations involving different segment of the gene can be found in patients with GATA2 deficiency, the use of homology-direct repair (HDR) as a correction mechanism, may be attempted using large HDR donor templates that cover the whole gene. Unfortunately, the low efficiency of HDR mediated repair combined with the decreased pool of HSCs present in patients, represent one of the major hurdles for gene therapy (53). In addition, the minimum level of chimerism to reverse the phenotype is still not clear. Nevertheless, a case of somatic genetic rescue (SGR) in an asymptomatic individual carrying a pathogenic GATA2 germline mutation was recently described. It was speculated that HSCs carrying the restored *GATA2* gene enjoyed a selective advantage over “diseased” cells, restoring normal bone marrow function (54, 55). This observation supports the hypothesis that even a small number of mutation-corrected HSCs might be sufficient to heal or at least mitigate the manifestations of GATA2 deficiency in affected patients.

## Conclusions

Germline mutations of *GATA2* cause a various and complex clinical phenotype, including immunodeficiency, bone marrow failure, lymphedema, deafness, and pulmonary dysfunction. Despite the emergent findings, the exact pathogenetic mechanism underlying these alterations is still not fully understood. Also, there are still many gaps regarding the optimal management of patients with GATA2 deficiency, highlighting the need for dedicated guidelines.

## Author Contributions

All authors listed have made a substantial, direct, and intellectual contribution to the work, and approved it for publication.

## Conflict of Interest

The authors declare that the research was conducted in the absence of any commercial or financial relationships that could be construed as a potential conflict of interest.

## Publisher’s Note

All claims expressed in this article are solely those of the authors and do not necessarily represent those of their affiliated organizations, or those of the publisher, the editors and the reviewers. Any product that may be evaluated in this article, or claim that may be made by its manufacturer, is not guaranteed or endorsed by the publisher.
